# Association between lactate dehydrogenase-to-albumin ratio and short-term mortality in critically ill patients with urosepsis: evidence from dual retrospective cohorts

**DOI:** 10.3389/fcimb.2026.1891664

**Published:** 2026-09-16

**Authors:** Guangyou Yang, Kunyuan Huang, Haoming Luo, Xinxin Peng

**Affiliations:** Department of Urology, Xingyi People’s Hospital Affiliatedo Guizhou Medical University, Xing Yi, China

**Keywords:** lactate dehydrogenase-to-albumin ratio, retrospective cohort study, risk prediction model, short-term mortality, urosepsis

## Abstract

**Background:**

The lactate dehydrogenase-to-albumin ratio (LDAR) reflects tissue damage, inflammation, and nutritional status. Its prognostic value in urosepsis remains unexplored. We evaluated LDAR’s association with short-term mortality and developed an LDAR-based prediction model.

**Methods:**

This retrospective dual-cohort study used MIMIC-IV (n=3,060) and an external cohort (n=370). Exposure was log_2_-transformed LDAR (logLDAR); outcomes were 28-day intensive care unit (ICU) and in-hospital mortality. Cox regression with three adjustment models, restricted cubic splines (RCS), and Kaplan-Meier curves assessed associations; Fine-Gray models addressed competing risks. DeLong tests compared area under the curve (AUC) of six conventional scores with and without logLDAR. Net reclassification improvement (NRI) and integrated discrimination improvement (IDI) quantified incremental value of adding logLDAR; Machine learning selected predictors for a parsimonious model validated internally and externally. decision curve analysis (DCA) evaluated the 7-variable model’s utility.

**Results:**

Among 3,060 patients, higher logLDAR was associated with significantly elevated 28-day ICU (23.4% vs. 9.02%) and in-hospital (22.1% vs. 8.24%) mortality (both P<0.001). In the primary multivariable-adjusted model, per-unit increases in logLDAR yielded hazard ratios (HRs) of 1.26 (95% CI: 1.17–1.36) for ICU mortality and 1.35 (95% CI: 1.25–1.45) for in-hospital mortality. Compared with the lowest tertile, the highest tertile showed HRs of 1.79 (1.40–2.30) and 2.29 (1.77–2.96), respectively. RCS analysis revealed linear dose-response relationships (P<0.001), and the Fine-Gray model confirmed robust associations (subdistribution hazard ratio (sHR)=1.36, 1.27–1.46). Adding logLDAR significantly improved the AUCs of all six conventional scores (DeLong P<0.01), with NRI/IDI showing substantial reclassification improvement (P<0.001). A parsimonious 7-variable model achieved AUCs of 0.674, 0.677, and 0.783 in the internal training, internal validation, and external cohorts, respectively, with adequate calibration (Hosmer-Lemeshow P > 0.05) and DCA demonstrating net clinical benefit. These findings were fully validated in an external real-world cohort.

**Conclusions:**

LDAR independently predicts short-term mortality in critically ill patients with urosepsis, supplementing conventional severity scores. The parsimonious risk model showed moderate performance with acceptable cross-cohort generalizability.

## Introduction

Sepsis is a life-threatening organ dysfunction caused by a dysregulated host response to infection, representing a leading cause of mortality among non-cardiac intensive care unit (ICU) patients (Cheng et al., 2025; Singer et al., 2026). Although lower respiratory tract infections remain the predominant source, the incidence of sepsis secondary to urinary tract infection (UTI) is escalating at an alarming rate (Dreger et al., 2015; GBD 2021 Global Sepsis Collaborators, 2025; Morrissey et al., 2025). This trend is further compounded by the increasing volume of urological procedures, the growing challenge of antimicrobial resistance, and an aging population with multiple comorbidities (Ryan et al., 2020; Dickson et al., 2024). Together, these converging factors underscore an urgent clinical need: the identification of reliable, rapidly obtainable biomarkers to facilitate early risk stratification and guide timely therapeutic intervention in patients with urosepsis.

In this context, two routine laboratory parameters—serum albumin (ALB) and lactate dehydrogenase (LDH)—have individually drawn considerable attention. ALB, the most abundant plasma protein, serves a dual role as both a nutritional marker and a negative acute-phase reactant that mirrors the intensity of systemic inflammation (Don and Kaysen, 2004; Linciano et al., 2022; Brahma et al., 2024). Indeed, hypoalbuminemia has been consistently linked to functional decline, disease progression, and increased all-cause mortality across diverse patient populations (Jellinge et al., 2014; Morotti et al., 2017; Seidu et al., 2020). Conversely, LDH, a cytosolic enzyme catalyzing the interconversion of lactate and pyruvate, rises markedly under conditions of tissue hypoxia, cellular necrosis, or hypoperfusion, rendering it a sensitive—though non-specific—indicator of cellular injury and disease severity in sepsis (Duman et al., 2016; Khan et al., 2020; Farhana and Lappin, 2023; Zhou et al., 2025; Ge et al., 2026). Nevertheless, when deployed in isolation, neither biomarker adequately captures the multifaceted pathophysiology of critical illness.

The LDH-to-albumin ratio (LDAR), by combining these complementary parameters, offers a composite measure that simultaneously reflects systemic organ damage, inflammatory-nutritional imbalance, oxidative stress, and tissue hypoxia. Emerging evidence has established LDAR as a robust prognostic indicator across various critical care settings, including heart failure, pulmonary embolism, and sepsis-associated acute kidney injury (Tu et al., 2026; Liang et al., 2023; Guan et al., 2024; Hu and Zhou, 2024). However, while other ratio-based biomarkers have been explored in urosepsis (Liu et al., 2026; Nie et al., 2026; Yu et al., 2026), the utility of LDAR in this population remains unexplored. This is a notable gap, because urosepsis exhibits distinct pathophysiological features—direct renal tubular injury from ascending infection, potential post-renal obstruction leading to pressure nephropathy, and a gradient of tissue damage from lower UTI to bacteremia—all of which may render LDAR particularly informative in this population (Petrosillo et al., 2020; Zhou et al., 2026). Therefore, the present study was designed to systematically evaluate the independent predictive value of LDAR for short-term mortality in critically ill patients with urosepsis, leveraging a large public database for discovery and an independent real-world cohort for rigorous external validation.

## Materials and methods

### Data sources

This study employed two retrospective cohorts. The internal discovery cohort was derived from the Medical Information Mart for Intensive Care IV (MIMIC-IV) database, which contains comprehensive clinical data from 196,527 adult patients admitted to Beth Israel Deaconess Medical Center between 2008 and 2019 (Ulrich et al., 2021). Because the analysis used only de-identified information, institutional review board (IRB) approval was not required. The external validation cohort comprised patients with urosepsis admitted to the Department of Urology and Critical Care Medicine at Xingyi People’s Hospital Affiliated to Guizhou Medical University between January 2020 and January 2025. The external cohort protocol was reviewed and approved by the hospital’s ethics committee, and informed consent was waived due to the retrospective design.

### Study population

Patients admitted to the ICU with sepsis secondary to UTI were enrolled. Sepsis was defined according to the Sepsis-3 international consensus criteria (Wei et al., 2024; Wei et al., 2025). In the MIMIC-IV cohort, urosepsis was identified by the coexistence of sepsis (per Sepsis-3 criteria) and a UTI diagnosis (ICD codes), with stringent exclusion of other potential infection foci (e.g., pneumonia, intra-abdominal infection, or non-urinary bloodstream infection). In the external validation cohort, diagnosis required: (1) Sepsis-3 criteria; (2) typical clinical manifestations of UTI (fever, dysuria, flank pain, frequency or urgency); (3) positive urine culture (≥10^5^ CFU/mL for midstream clean-catch specimens); and (4) exclusion of other infection sources by comprehensive clinical and microbiological assessment. Inclusion criteria for both cohorts were: (1) age ≥18 years and (2) complete LDH and ALB measurements available at ICU admission.

### Data extraction and handling of missing values

Data extraction from MIMIC-IV was performed using PostgreSQL (13.7.2), Navicat Premium (16.0), and structured query language (SQL). Extracted variables encompassed six domains: demographic characteristics, underlying comorbidities, vital signs, laboratory test results, disease severity scores, and therapeutic interventions. A complete abbreviation list is provided in **Supplementary Table 1**. The primary exposure was LDAR, calculated as LDH (U/L)/ALB (g/dL). Because the distribution of LDAR was heavily right-skewed (skewness=9.69, kurtosis=155.50; Shapiro-Wilk W = 0.313, P<2.2×10^-^¹^6^), we applied a log_2_ transformation (logLDAR). This transformation normalized the distribution (post-transformation: skewness=1.57, kurtosis=3.92) and offered a clinically intuitive interpretation, where each one-unit increase corresponds to a doubling of the raw LDAR. Variables with >30% missingness were excluded from the primary analysis. Missing values in the remaining variables were imputed using multiple imputation with the ‘mice’ package (version 3.16.0) in R, incorporating all variables included in the primary analyses to strengthen the plausibility of the missing at random (MAR) assumption. Detailed proportions of missing data and the core imputation code are provided in the **Supplementary Materials**.

### Association between logLDAR and clinical outcomes

Cox proportional hazards regression was used to evaluate associations between logLDAR and clinical endpoints, with the proportional hazards assumption verified using Schoenfeld residuals. Three stepwise-adjusted models were constructed: Model 1 (unadjusted); Model 2 (adjusted for age, sex, race, weight, and baseline comorbidities: acute kidney injury (AKI), chronic kidney disease (CKD), hyperlipidemia (HLD), heart failure (HF), myocardial infarction (MI), and chronic obstructive pulmonary disease (COPD)); Model 3 (additionally adjusted for key laboratory indices and treatment measures with variance inflation factor (VIF) <5, excluding albumin and LDH to avoid overadjustment and multicollinearity). Model 2, which includes only pre-treatment covariates, was prespecified as the primary adjustment model to minimize potential mediator and collider bias from post-exposure interventions; Model 3 served as a sensitivity analysis. Restricted cubic splines (RCS) with knots at the 5th, 35th, 65th, and 95th percentiles explored non-linear relationships. Kaplan-Meier curves with log-rank tests further validated the association. For the external cohort, given the limited number of events (n=44), a parsimonious model adjusting only for Age and AKI was employed to achieve an events-per-variable (EPV) ratio of 14.7, exceeding the recommended minimum of 10 and thus avoiding overfitting. To account for the competing risk of alive ICU discharge, Fine-Gray subdistribution hazard models were fitted, and cumulative incidence function (CIF) plots were generated.

### Incremental effect of logLDAR

LogLDAR was added to six established severity scoring systems: APACHE II, APS III, Charlson comorbidity index, OASIS, SAPS II, and SOFA. Multivariate logistic regression models were developed, and receiver operating characteristic (ROC) curves were plotted. The DeLong test compared AUCs before and after adding logLDAR. NRI and IDI were calculated to quantify the improvement in risk reclassification.

### Subgroup and interaction analyses

Pre-specified subgroup analyses stratified by age, sex, race, and key comorbidities (hypertension, AKI, diabetes mellitus, CKD, HLD, HF, MI, IHD, and COPD) were performed to assess the robustness of the association. Interaction P-values were calculated and adjusted for multiple comparisons using the Benjamini-Hochberg false discovery rate (FDR) method.

### Clinical value of logLDAR

In the MIMIC-IV cohort, the optimal logLDAR cut-off for predicting 28-day ICU mortality was determined by maximizing the Youden index (sensitivity + specificity – 1). The sensitivity, specificity, and Youden index at this threshold were reported to evaluate its discriminative ability.

### Screening of important prognostic features

The internal cohort was randomly split into a training set (70%) and a validation set (30%). In the training set, four machine learning algorithms—random forest (100 trees, max depth 3, min node size 1), gradient boosting machine (GBM) (Bernoulli loss, shrinkage 0.1, 100 iterations, interaction depth 3), Lasso regression (10-fold cross-validation, lambda.1se), and support vector machine (SVM) (radial basis kernel, C = 1, gamma=0.01)—were used for joint variable screening. Variables identified as important by all four algorithms were designated as core predictors. Five-fold and ten-fold stratified cross-validation were performed to evaluate and quantify the degree of overfitting.

### Risk prediction modeling and validation

Core predictors were incorporated into a multivariate logistic regression model. The risk score was calculated as: risk score = intercept + Σ(β_i_ × variable_i_). Model performance was evaluated using ROC curves and AUCs, with pairwise DeLong tests against conventional severity scores. Calibration was assessed using the Hosmer-Lemeshow goodness-of-fit test (with 10 groups), calibration plots comparing predicted versus observed probabilities across deciles, and Brier scores as a summary measure. DCA quantified the clinical net benefit of the model across a range of threshold probabilities. All statistical analyses were performed using R software (version 4.4.3), with a two-sided P<0.05 considered statistically significant.

## Results

### Baseline characteristics

A total of 3,060 critically ill patients with urosepsis were included (mean age 70.5 years; 43.4% male). Baseline characteristics stratified by logLDAR tertiles are shown in **Table 1**. Patients in the highest tertile exhibited a substantially more compromised baseline status compared with those in the lowest tertile. This included higher proportions of AKI (70.5% vs. 55.2%), HF (39.4% vs. 36.4%), and IHD (40.0% vs. 32.0%); more frequent use of vasopressors (71.2% vs. 51.2%), glucocorticoids (35.5% vs. 28.7%), and continuous renal replacement therapy (16.6% vs. 6.96%); lower systolic blood pressure; and elevated heart and respiratory rates. Laboratory findings revealed higher levels of RDW, potassium, lactate, INR, PTT, PT, creatinine, and urea, alongside lower albumin, CO_2_CP, and pH in the high logLDAR group. All clinical severity scores (SOFA, APS III, SAPS II, OASIS, and APACHE II) were positively correlated with logLDAR level. Most notably, 28-day ICU mortality (23.4% vs. 9.02%) and in-hospital mortality (22.1% vs. 8.24%) were significantly higher in the high logLDAR group (both P<0.001).

**Table 1:** Baseline characteristics of critically ill patients with urosepsis stratified by tertiles of logLDAR.

Characteristics	All patients (N = 3060)	Low tertile (N = 1020)	Moderate tertile (N = 1020)	High tertile (N = 1020)	P value
LDAR	171 (317)	62.4 (12.5)	102 (13.8)	348 (503)	0.000
logLDAR	6.84 (1.03)	5.93 (0.32)	6.66 (0.19)	7.93 (0.98)	0.000
Age	70.5 (15.1)	71.0 (14.7)	71.6 (14.9)	69.0 (15.5)	<0.001
Gender:	1329 (43.4%)	445 (43.6%)	454 (44.5%)	430 (42.2%)	0.556
Race:	1981 (64.7%)	683 (67.0%)	676 (66.3%)	622 (61.0%)	0.008
Weight	82.0 (26.0)	82.1 (25.6)	80.7 (25.2)	83.3 (27.1)	0.076
HPY:	1077 (35.2%)	387 (37.9%)	349 (34.2%)	341 (33.4%)	0.075
AKI:	1918 (62.7%)	563 (55.2%)	636 (62.4%)	719 (70.5%)	<0.001
CKD:	889 (29.1%)	299 (29.3%)	306 (30.0%)	284 (27.8%)	0.548
DM:	1155 (37.7%)	387 (37.9%)	399 (39.1%)	369 (36.2%)	0.386
HLD:	1074 (35.1%)	376 (36.9%)	363 (35.6%)	335 (32.8%)	0.151
HF:	1201 (39.2%)	371 (36.4%)	428 (42.0%)	402 (39.4%)	0.035
MI:	316 (10.3%)	55 (5.39%)	110 (10.8%)	151 (14.8%)	<0.001
IHD:	1126 (36.8%)	326 (32.0%)	392 (38.4%)	408 (40.0%)	<0.001
COPD:	492 (16.1%)	189 (18.5%)	156 (15.3%)	147 (14.4%)	0.029
SOFA	6.90 (3.46)	6.06 (3.12)	6.71 (3.24)	7.92 (3.72)	<0.001
APSIII	58.9 (21.1)	54.1 (18.7)	57.7 (19.8)	65.0 (23.0)	<0.001
SAPSII	45.0 (13.4)	42.5 (12.3)	44.5 (12.7)	48.2 (14.5)	<0.001
OASIS	36.0 (8.33)	34.7 (7.94)	36.0 (7.96)	37.2 (8.88)	<0.001
Charlson	6.31 (2.91)	6.19 (2.87)	6.43 (2.82)	6.31 (3.03)	0.165
APACHEII	21.8 (7.00)	20.5 (6.42)	21.7 (6.85)	23.2 (7.44)	<0.001
HR	91.5 (21.3)	88.0 (20.4)	91.4 (20.6)	94.9 (22.2)	<0.001
NBPS	120 (25.8)	123 (25.3)	120 (25.6)	118 (26.2)	<0.001
NBPD	67.6 (19.8)	67.2 (19.3)	67.6 (19.8)	67.8 (20.3)	0.788
RR	20.2 (6.39)	19.6 (6.11)	20.5 (6.71)	20.5 (6.31)	0.002
Spo2	96.5 (4.68)	96.8 (4.25)	96.6 (4.14)	96.1 (5.51)	0.004
HCT	31.3 (6.64)	31.4 (6.39)	31.5 (6.69)	31.1 (6.84)	0.392
Hb	10.1 (2.20)	10.1 (2.14)	10.2 (2.22)	10.1 (2.24)	0.457
PLT	200 (117)	205 (108)	207 (119)	189 (123)	0.001
RDW	16.1 (2.61)	15.8 (2.39)	16.0 (2.54)	16.4 (2.86)	<0.001
RBC	3.40 (0.78)	3.42 (0.76)	3.41 (0.77)	3.37 (0.80)	0.242
WBC	13.9 (14.8)	12.2 (17.0)	13.7 (10.3)	15.7 (15.9)	<0.001
ALB	2.90 (0.62)	3.20 (0.55)	2.86 (0.57)	2.65 (0.60)	<0.001
GP	15.7 (4.76)	15.2 (4.34)	15.4 (4.55)	16.6 (5.23)	<0.001
Ca	8.26 (0.98)	8.47 (0.94)	8.24 (0.89)	8.07 (1.07)	<0.001
Cl	104 (8.02)	104 (7.64)	104 (8.03)	104 (8.38)	0.233
Glu	133 (105,175)	130 [102;166]	133 [106;175]	137 [106;187]	0.004
K	4.23 (0.81)	4.20 (0.74)	4.15 (0.78)	4.33 (0.88)	<0.001
Na	139 (6.86)	138 (6.41)	139 (6.96)	138 (7.16)	0.015
CO2CP	23.9 (6.55)	24.6 (6.86)	24.3 (6.36)	22.7 (6.24)	<0.001
LAC	2.40 (1.99)	1.89 (1.40)	2.26 (1.67)	3.04 (2.53)	<0.001
PCO2	42.2 (12.7)	43.1 (13.8)	42.2 (12.3)	41.2 (11.9)	0.004
PH	7.35 (0.11)	7.35 (0.10)	7.36 (0.10)	7.33 (0.11)	<0.001
PO2	116 (95.3)	115 (93.7)	115 (94.1)	118 (98.3)	0.705
INR	1.40 [1.20;1.70]	1.30 [1.10;1.70]	1.40 [1.20;1.70]	1.50 [1.20;1.90]	<0.001
PT	15.0 [13.0;18.9]	14.4 [12.6;18.0]	14.9 [12.9;18.4]	15.9 [13.5;20.3]	<0.001
PTT	32.4 [27.8;41.2]	31.1 [27.4;39.3]	32.2 [27.5;40.8]	33.9 [28.6;44.2]	<0.001
ALT	25.0 [15.0;53.0]	18.0 [12.0;28.2]	25.0 [15.0;47.0]	44.0 [22.8;130]	<0.001
AST	39.0 [23.0;86.0]	25.0 [18.0;39.2]	37.0 [23.0;68.0]	86.0 [42.0;258]	<0.001
TB	0.70 [0.40;1.40]	0.50 [0.30;1.10]	0.60 [0.40;1.40]	0.90 [0.50;2.10]	<0.001
CREA	1.30 [0.80;2.20]	1.20 [0.80;2.10]	1.30 [0.80;2.10]	1.40 [0.90;2.40]	<0.001
UREA	29.0 [18.0;49.0]	27.0 [17.0;46.0]	28.0 [17.0;49.0]	32.0 [19.0;54.0]	<0.001
LDH	284 [214;409]	198 [165;227]	286 [242;333]	510 [399;799]	0.000
SA:	2052 (67.1%)	610 (59.8%)	689 (67.5%)	753 (73.8%)	<0.001
VP:	1908 (62.4%)	522 (51.2%)	660 (64.7%)	726 (71.2%)	<0.001
GC:	995 (32.5%)	293 (28.7%)	340 (33.3%)	362 (35.5%)	0.004
Ventilation:	2637 (86.2%)	856 (83.9%)	893 (87.5%)	888 (87.1%)	0.036
CRRT:	321 (10.5%)	71 (6.96%)	81 (7.94%)	169 (16.6%)	<0.001
Hosp day	13.2 [7.59;23.2]	12.2 [6.91;21.7]	13.5 [7.80;22.8]	14.2 [7.91;24.8]	0.002
ICU day	3.97 [1.98;8.79]	3.44 [1.82;7.59]	4.04 [1.95;8.23]	4.67 [2.32;10.2]	<0.001
Hosp dead	437 (14.3%)	84 (8.24%)	128 (12.5%)	225 (22.1%)	<0.001
ICU dead	471 (15.4%)	92 (9.02%)	140 (13.7%)	239 (23.4%)	<0.001

Data are presented as mean (SD), median [IQR], or n (%). P-values were calculated using ANOVA, Kruskal-Wallis test, chi-squared test, or Fisher’s exact test as appropriate. AKI, acute kidney injury; ALB, albumin; ALT, alanine aminotransferase; APACHE II, Acute Physiology and Chronic Health Evaluation II; APS III, Acute Physiology Score III; AST, aspartate aminotransferase; Ca, calcium; CI, confidence interval; CKD, chronic kidney disease; Cl, chloride; COPD, chronic obstructive pulmonary disease; CO_2_CP, total carbon dioxide combining power; CREA, creatinine; CRRT, continuous renal replacement therapy; DM, diabetes mellitus; GA, gap of anion; GC, glucocorticoid; Glu, glucose; Hb, hemoglobin; HCT, hematocrit; HF, heart failure; HLD, hyperlipidemia; HPY, hypertension; HR, heart rate; ICU, intensive care unit; IHD, ischemic heart disease; INR, international normalized ratio; K, potassium; LAC, lactate; LDH, lactate dehydrogenase; MI, myocardial infarction; Na, sodium; NBPD, non-invasive blood pressure diastolic; NBPS, non-invasive blood pressure systolic; OASIS, Oxford Acute Severity of Illness Score; PCO_2_, partial pressure of carbon dioxide; PH, potential of hydrogen; PLT, platelet count; PO_2_, partial pressure of oxygen; PT, prothrombin time; PTT, partial thromboplastin time; RBC, red blood cell count; RDW, red cell distribution width; RR, respiratory rate; SA, sedation-analgesia; SAPS II, Simplified Acute Physiology Score II; SOFA, Sequential Organ Failure Assessment; SpO_2_, oxygen saturation; TB, total bilirubin; UREA, urea; VP, vasopressor; WBC, white blood cell count.

### Association between logLDAR and short-term mortality

Multivariable Cox regression analyses demonstrated that logLDAR, as a continuous variable, was significantly and positively associated with both ICU mortality (primary Model 2 HR = 1.26, 95% CI: 1.17–1.36, P<0.001) and in-hospital mortality (Model 2 HR = 1.35, 95% CI: 1.25–1.45, P<0.001). These results remained robust across Model 1 and the sensitivity Model 3 (**Table 2** and **3**). When analyzed categorically by tertiles, the highest tertile consistently showed significantly elevated mortality risk (ICU: HR = 1.79, 1.40–2.30; in-hospital: HR = 2.29, 1.77–2.96; all P<0.001), whereas the medium tertile demonstrated a non-significant association with ICU mortality (HR = 1.24, 0.95–1.61, P = 0.12) but a significant association with in-hospital mortality (HR = 1.36, 1.03–1.79, P = 0.030). RCS curves confirmed a linear dose-response relationship for ICU mortality (P for non-linearity=0.089) and a monotonically increasing relationship for in-hospital mortality (P for non-linearity=0.007) (**Figures 1A–C**). Kaplan-Meier survival curves revealed progressively shorter survival times with increasing LDAR tertiles (**Figures 1D–F**).

**Table 2:** Association between logLDAR and 28-day ICU mortality in critically ill patients with urosepsis: Cox proportional hazards regression models.

Characteristic	Model1			Model2			Model3		
	HR	95% CI	P value	HR	95% CI	P value	HR	95% CI	P value
Log LDAR	1.27	1.19, 1.37	<0.001	1.26	1.17, 1.36	<0.001	1.29	1.18, 1.42	<0.001
LDAR group									
Low	Ref	Ref		Ref	Ref		Ref	Ref	
Moderate	1.36	1.04, 1.76	0.023	1.24	0.95, 1.61	0.12	1.17	0.90, 1.53	0.25
High	1.98	1.56, 2.52	<0.001	1.79	1.40, 2.30	<0.001	1.74	1.34, 2.25	<0.001

CI , Confidence Interval, HR , Hazard Ratio.

**Table 3:** Association between logLDAR and 28-day in-hospital mortality in critically ill patients with urosepsis: Cox proportional hazards regression models.

Characteristic	Model1			Model2			Model3		
	HR	95% CI	P value	HR	95% CI	P value	HR	95% CI	P value
Log LDAR	1.34	1.25, 1.44	<0.001	1.35	1.25, 1.45	<0.001	1.38	1.26, 1.52	<0.001
LDAR group									
Low	Ref	Ref		Ref	Ref		Ref	Ref	
Moderate	1.44	1.09, 1.89	0.010	1.36	1.03, 1.79	0.030	1.29	0.97, 1.70	0.08

CI , Confidence Interval, HR , Hazard Ratio.

**Figure 1:**
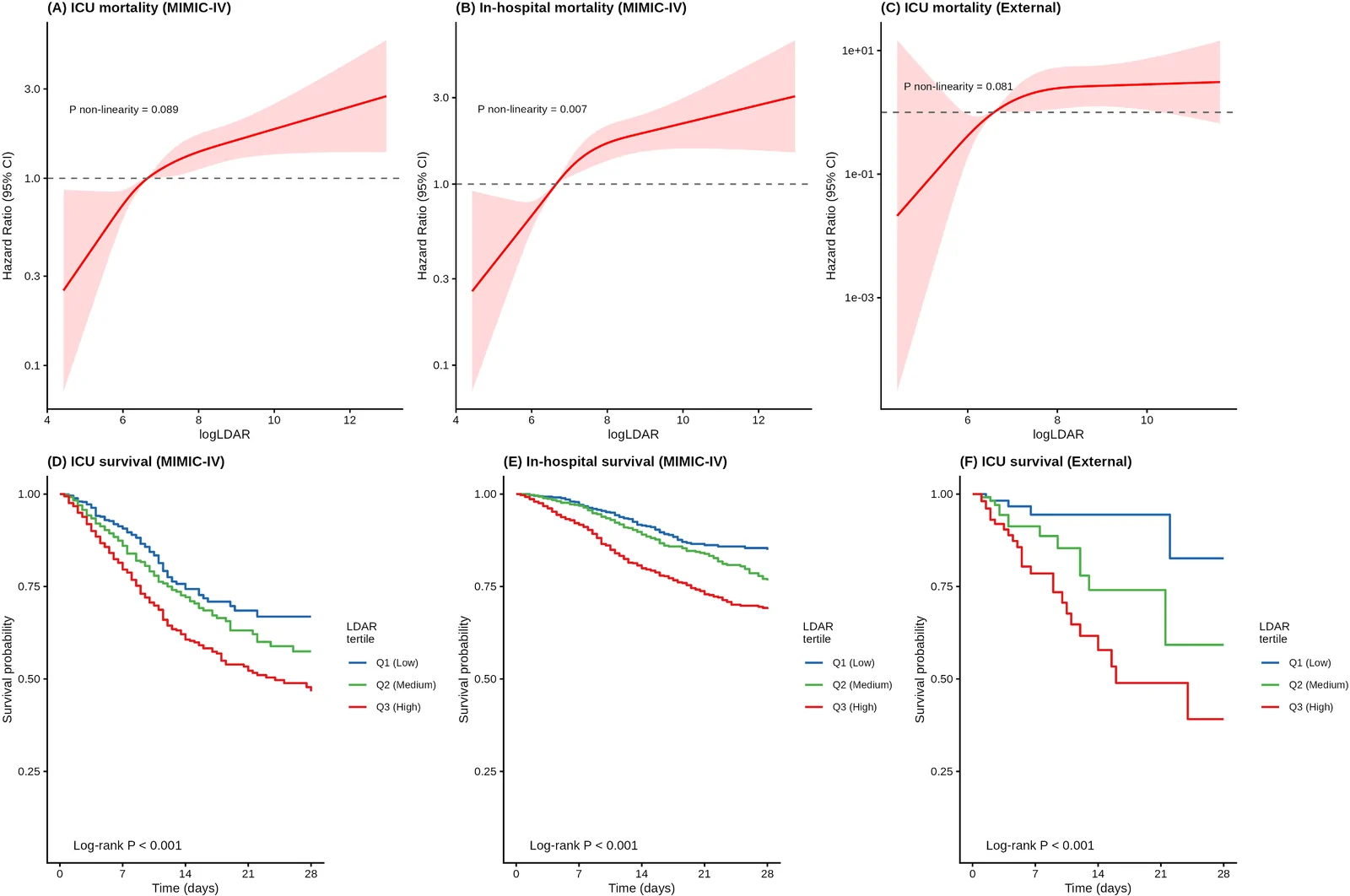
Dose-response relationship and Kaplan-Meier survival curves for logLDAR and short-term mortality. **(A–C)** Restricted cubic spline plots for 28-day ICU mortality (internal cohort), in-hospital mortality (internal cohort), and ICU mortality (external cohort), adjusted for Model 2 covariates. **(D–F)** Kaplan-Meier curves stratified by logLDAR tertiles for ICU survival (internal), in-hospital survival (internal), and ICU survival (external). Log-rank P-values are shown.

Accounting for the competing risk of alive ICU discharge, Fine-Gray subdistribution hazard models (**Table 4**) yielded results consistent with the primary Cox regression. In the derivation cohort, the continuous logLDAR sHR was 1.36 (1.27–1.46), and the high- versus low-tertile sHR was 2.59 (2.02–3.31); in the external cohort, the continuous sHR was 1.53 (1.29–1.81) and the high-versus-low tertile sHR was 6.58 (2.46–17.60). CIF curves are presented in **Figure 2**. Furthermore, given the pathophysiological link between LDH and lactate (both reflecting tissue hypoxia), a sensitivity analysis excluding lactate from Model 3 was performed. The logLDAR HR for ICU mortality remained virtually unchanged (with lactate: 1.29; without lactate: 1.30), confirming the robustness and reliability of the association.

**Table 4:** Fine-Gray subdistribution hazard model for the association between logLDAR and 28-day ICU mortality accounting for the competing risk of alive ICU discharge.

Cohort	Variable	sHR	95% CI	P value
Derivation	logLDAR (continuous)	1.36	1.27-1.46	<0.001
Derivation	Moderate vs. Low	1.47	1.13-1.92	0.004
Derivation	High vs. Low	2.59	2.02-3.31	<0.001
External	logLDAR (continuous)	1.53	1.29-1.81	<0.001
External	Moderate vs. Low	3.12	1.12-8.70	0.030
External	High vs. Low	6.58	2.46-17.60	<0.001

sHR , subdistribution hazard ratio from Fine-Gray competing risk model. Competing event = alive ICU discharge. Derivation cohort adjusted for the same covariates as Cox Model 2. External cohort adjusted for Age and AKI only (EPV = 14.7).

**Figure 2:**
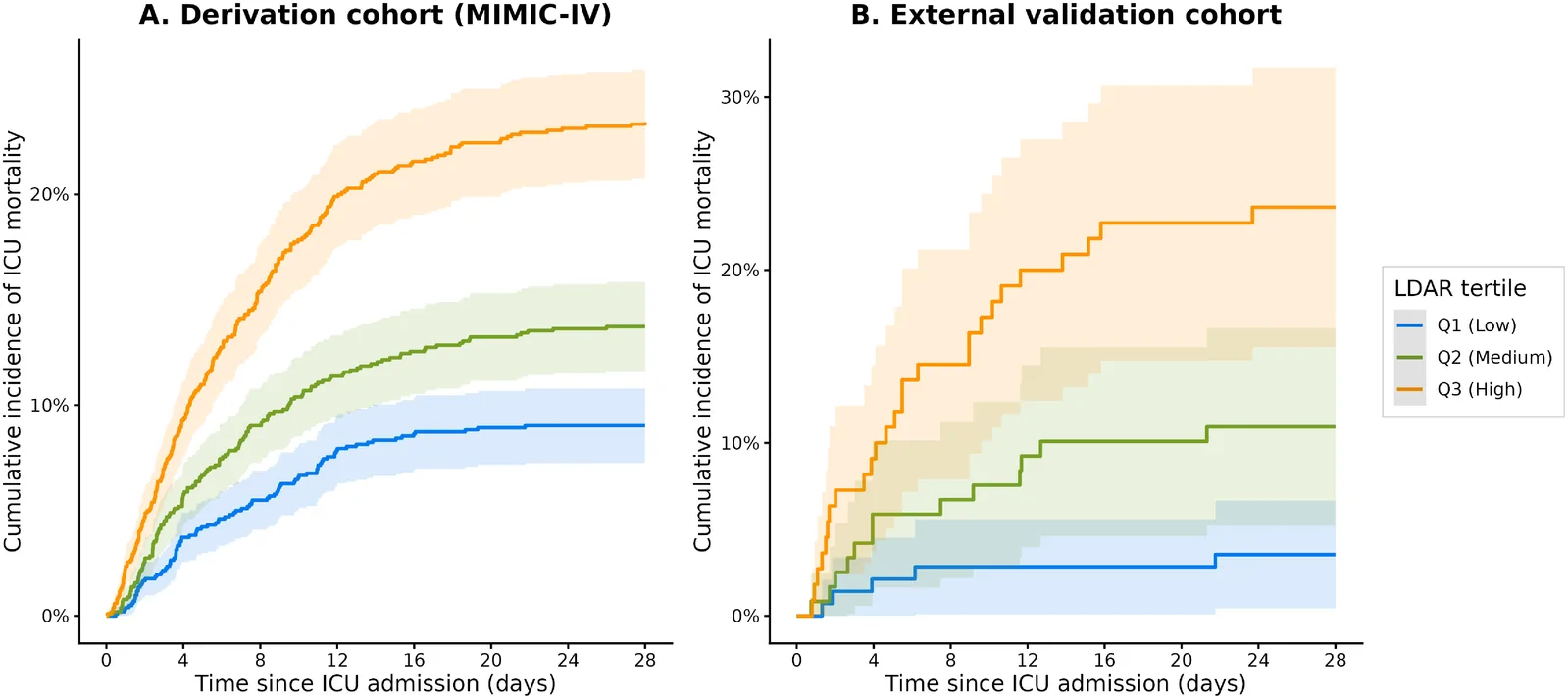
Cumulative incidence function curves from Fine-Gray competing risk models for 28-day ICU mortality, stratified by LDAR tertiles, in **(A)** the derivation cohort and **(B)** the external validation cohort. The competing event was alive ICU discharge.

### Incremental effect of logLDAR

Adding logLDAR to each of the six conventional severity scores significantly improved their AUCs for predicting both 28-day ICU and in-hospital mortality (DeLong test P<0.01 for all comparisons) (**Figure 3**). For ICU mortality, the AUC increments ranged from +0.026 for SAPS II (0.671 to 0.698) to +0.066 for the Charlson comorbidity index (0.593 to 0.659), with comparable improvements observed for in-hospital mortality. Furthermore, the addition of logLDAR to APACHE II, SOFA, and SAPS II yielded highly significant NRI and IDI values (all P<0.001), confirming that LDAR provides substantial risk reclassification beyond what traditional scoring systems alone can offer.

**Figure 3:**
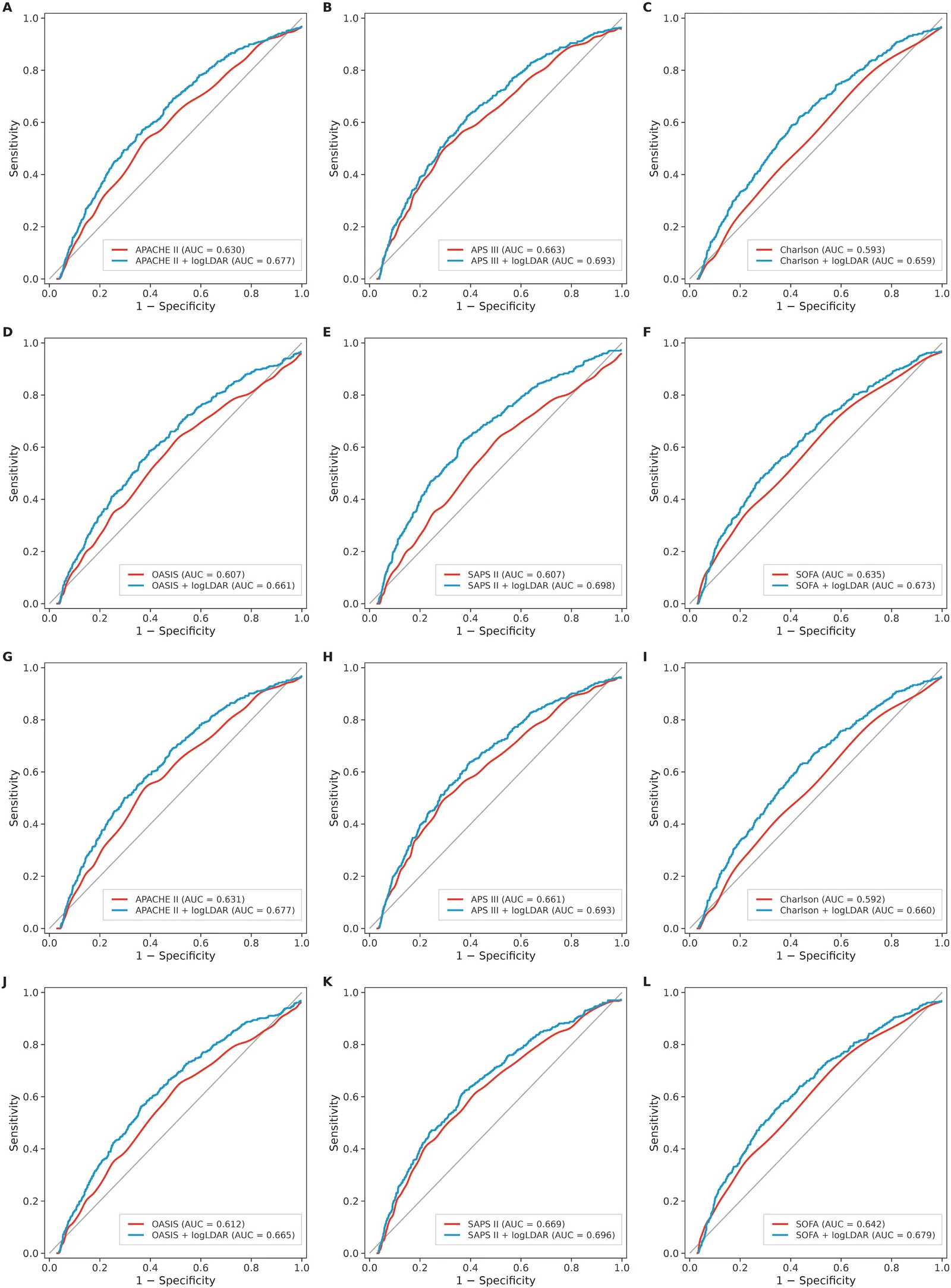
Incremental predictive value of adding logLDAR to six conventional severity scores. ROC curves and AUCs (with 95% CI) before (dashed) and after (solid) adding logLDAR for **(A–F)** 28-day ICU mortality and **(G–L)** 28-day in-hospital mortality. DeLong P<0.01 for all comparisons.

### Subgroup and interaction analyses

Stratified analyses by age, sex, race, and major comorbidities consistently demonstrated that the positive association between logLDAR and mortality remained stable across all predefined patient subsets. Although several unadjusted interaction P-values approached statistical significance—most notably AKI for ICU mortality (P = 0.049) and COPD (P = 0.038), age (P = 0.051), and hyperlipidemia (P = 0.053) for in-hospital mortality—none remained significant after Benjamini-Hochberg FDR correction (all FDR>0.05). These results indicate that the prognostic effect of logLDAR is broadly consistent across diverse patient populations, supporting its generalizability in clinical practice (**Supplementary Figures 1**, **2**).

### External cohort validation

The external validation cohort comprised 370 patients with a 28-day mortality rate of 11.89%. Consistent with the internal cohort findings, non-survivors exhibited significantly higher logLDAR levels than survivors (7.50 ± 1.16 vs. 6.68 ± 1.02, P<0.001) (**Supplementary Table 3**). In the parsimonious Cox model adjusted for Age and AKI, each unit increase in logLDAR remained independently associated with a 52% increase in 28-day mortality risk (HR = 1.52, 95% CI: 1.23–1.87, P<0.001). RCS analysis further validated a linear positive dose-response relationship (P<0.001, **Figure 1C**), and Kaplan-Meier curves demonstrated significantly shorter survival in the higher logLDAR group (**Figure 1F**). Collectively, these external data independently corroborate the primary findings from the MIMIC-IV cohort, reinforcing the robustness and cross-population validity of our observations.

### Clinical value of logLDAR

To enhance its clinical applicability, we determined the optimal logLDAR cut-off for predicting 28-day ICU mortality by maximizing the Youden index, yielding a threshold of 7.010. At this cut-off, the sensitivity was 51.0% and specificity 69.7% (Youden index=0.207). The moderate sensitivity precludes its use as a standalone rule-out screening tool; however, the relatively higher specificity suggests that an elevated logLDAR effectively “rules in” patients at genuinely increased risk, thereby supporting its clinical utility as an adjunctive early-warning signal. In practice, this threshold may assist clinicians in identifying individuals who warrant enhanced monitoring or earlier escalation of care, particularly when integrated with conventional severity scoring systems in a two-step risk-stratification approach.

### Development of a LDAR-associated risk prediction model

Given the moderate performance of LDAR as a standalone biomarker, we developed a parsimonious risk prediction model. In the internal training cohort, four machine learning algorithms were jointly used for variable screening. Random forest identified 7 variables with non-zero importance; GBM identified 46 variables (top 10 selected for cross-algorithm intersection); Lasso selected 15 variables; and SVM identified 49 variables (top 10 selected for intersection). The intersection of the four algorithms yielded seven core predictors: logLDAR, RDW, CO_2_CP, LAC, INR, TB, and UREA (**Figure 4**). The multivariate logistic regression model was formulated as:Risk score = -5.598 + 0.317×logLDAR + 0.070×RDW – 0.002×CO_2_CP + 0.034×LAC + 0.103×INR + 0.025×TB + 0.006×UREA.

**Figure 4:**
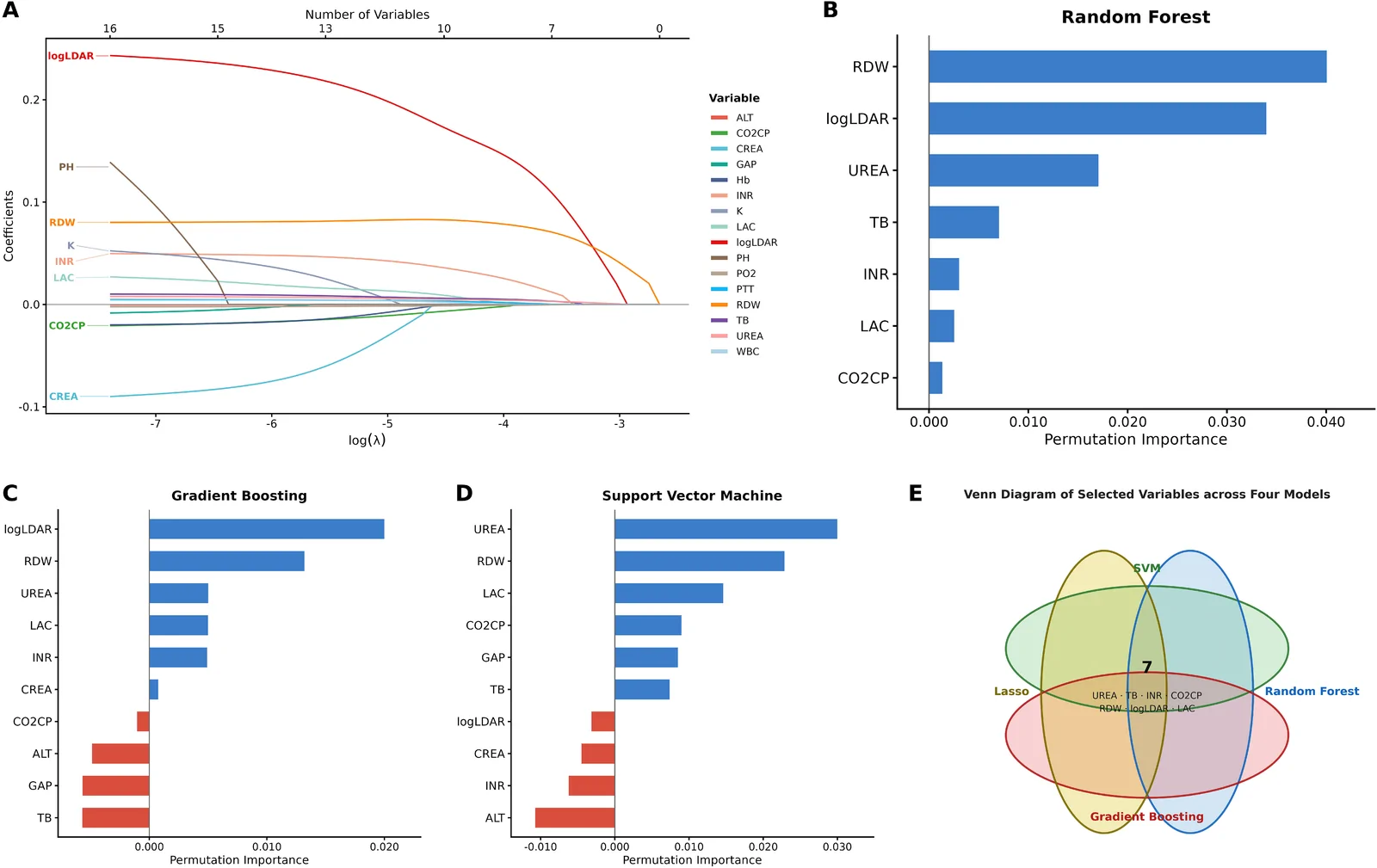
Machine learning selection of core predictors for 28-day ICU mortality. Variable importance from **(A)** random forest, **(B)** gradient boosting machine, **(C)** Lasso regression coefficients, and **(D)** support vector machine. **(E)** Venn diagram showing the intersection of seven core predictors (logLDAR, RDW, CO_2_CP, LAC, INR, TB, UREA) identified by all four algorithms.

This model achieved AUCs of 0.674, 0.677, and 0.783 in the internal training, internal validation, and external validation cohorts, respectively (**Figure 5**). In the training cohort, the model significantly outperformed SOFA (P = 0.014), OASIS (P<0.001), Charlson (P<0.001), and APACHE II (P = 0.034), while showing comparable performance to APS III (P = 0.727) and SAPS II (P = 0.961) (**Table 5**). In the external cohort, the model outperformed all conventional scores except OASIS. Cross-validation yielded mean AUCs of 0.668 ± 0.013 (5-fold) and 0.672 ± 0.043 (10-fold), consistent with the original split, indicating minimal overfitting. The Hosmer-Lemeshow test showed no significant lack of fit in any cohort (training P = 0.251, validation P = 0.155, external P = 0.121); Brier scores were 0.126, 0.116, and 0.096, respectively, indicating good predictive reliability (**Figure 6**). DCA demonstrated a superior net benefit across a wide range of threshold probabilities compared with logLDAR alone and conventional scores (**Figure 7**), supporting its potential clinical utility when used in conjunction with clinical judgment.

**Figure 5:**
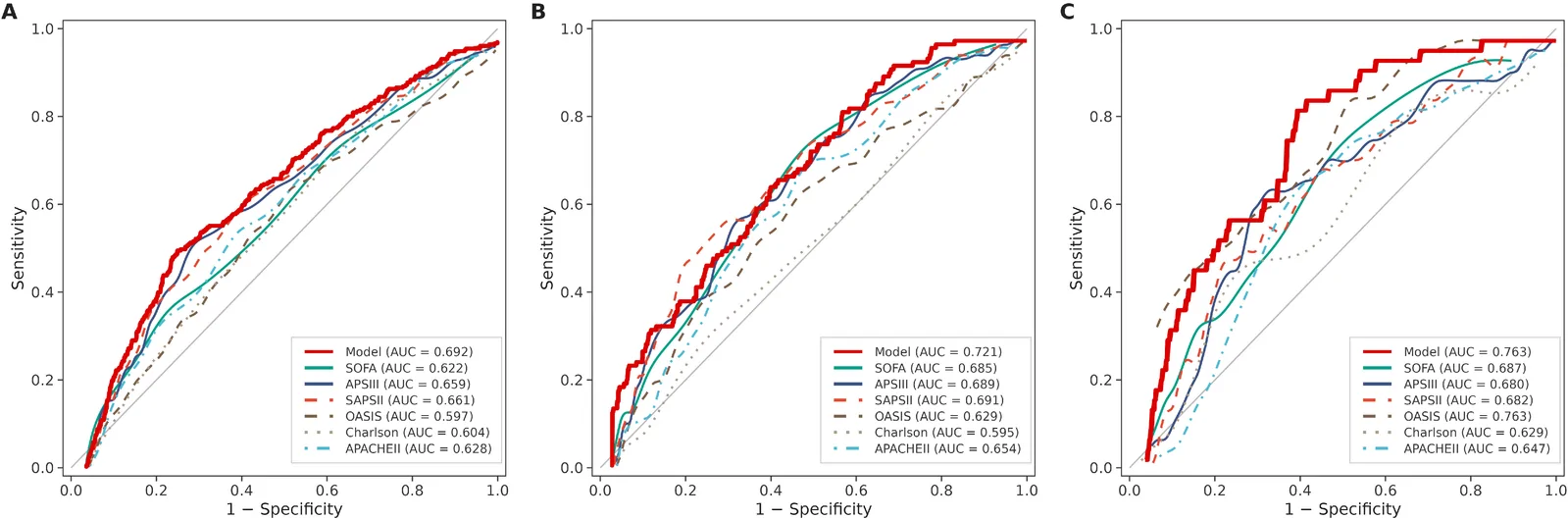
ROC curves of the 7-variable logistic regression model for 28-day ICU mortality in **(A)** the internal training cohort (AUC = 0.674), **(B)** the internal validation cohort (AUC = 0.677), and **(C)** the external validation cohort (AUC = 0.783). Risk score formula is shown.

**Table 5:** DeLong test comparisons of the 7-variable risk model with conventional severity scores for predicting 28-day ICU mortality.

Cohort	Comparison	Model AUC	Score AUC	Difference	P value
Training	Model vs SOFA	0.674	0.632	0.042	0.014
Training	Model vs APS III	0.674	0.668	0.006	0.727
Training	Model vs SAPS II	0.674	0.673	0.001	0.961
Training	Model vs OASIS	0.674	0.594	0.080	<0.001
Training	Model vs Charlson	0.674	0.576	0.098	<0.001
Training	Model vs APACHE II	0.674	0.635	0.039	0.034
Validation	Model vs SOFA	0.677	0.642	0.035	0.208
Validation	Model vs APS III	0.677	0.650	0.027	0.319
Validation	Model vs SAPS II	0.677	0.665	0.012	0.709
Validation	Model vs OASIS	0.677	0.641	0.037	0.309
Validation	Model vs Charlson	0.677	0.636	0.042	0.230
Validation	Model vs APACHE II	0.677	0.615	0.062	0.041
External	Model vs SOFA	0.783	0.687	0.096	0.012
External	Model vs APS III	0.783	0.680	0.103	0.018
External	Model vs SAPS II	0.783	0.682	0.101	0.032
External	Model vs OASIS	0.783	0.763	0.020	0.676
External	Model vs Charlson	0.783	0.629	0.154	0.006
External	Model vs APACHE II	0.783	0.647	0.135	0.001

AUC , area under the receiver operating characteristic curve. P value from DeLong test for pairwise AUC comparison. Bold P values indicate statistical significance (P < 0.05).

**Figure 6:**
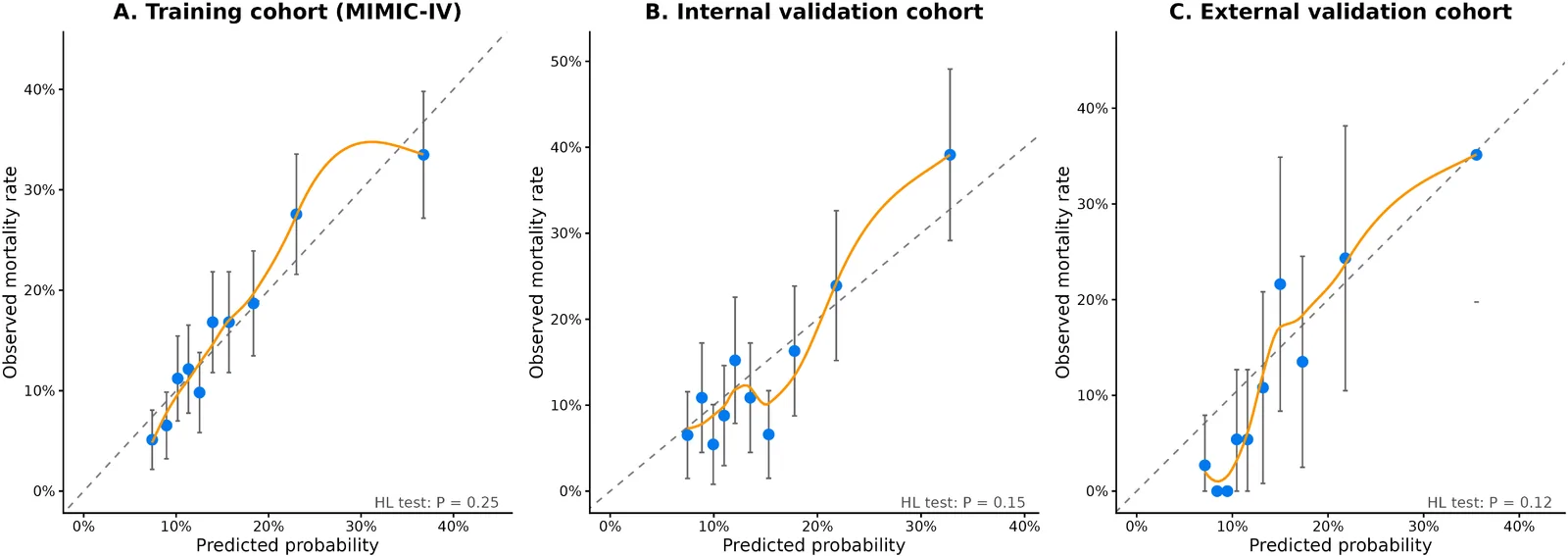
Calibration plots of the 7-variable risk prediction model in **(A)** the internal training, **(B)** internal validation, and **(C)** external validation cohorts. Diagonal dashed line indicates perfect calibration. Hosmer-Lemeshow P-values: 0.251, 0.155, and 0.121, respectively.

**Figure 7:**
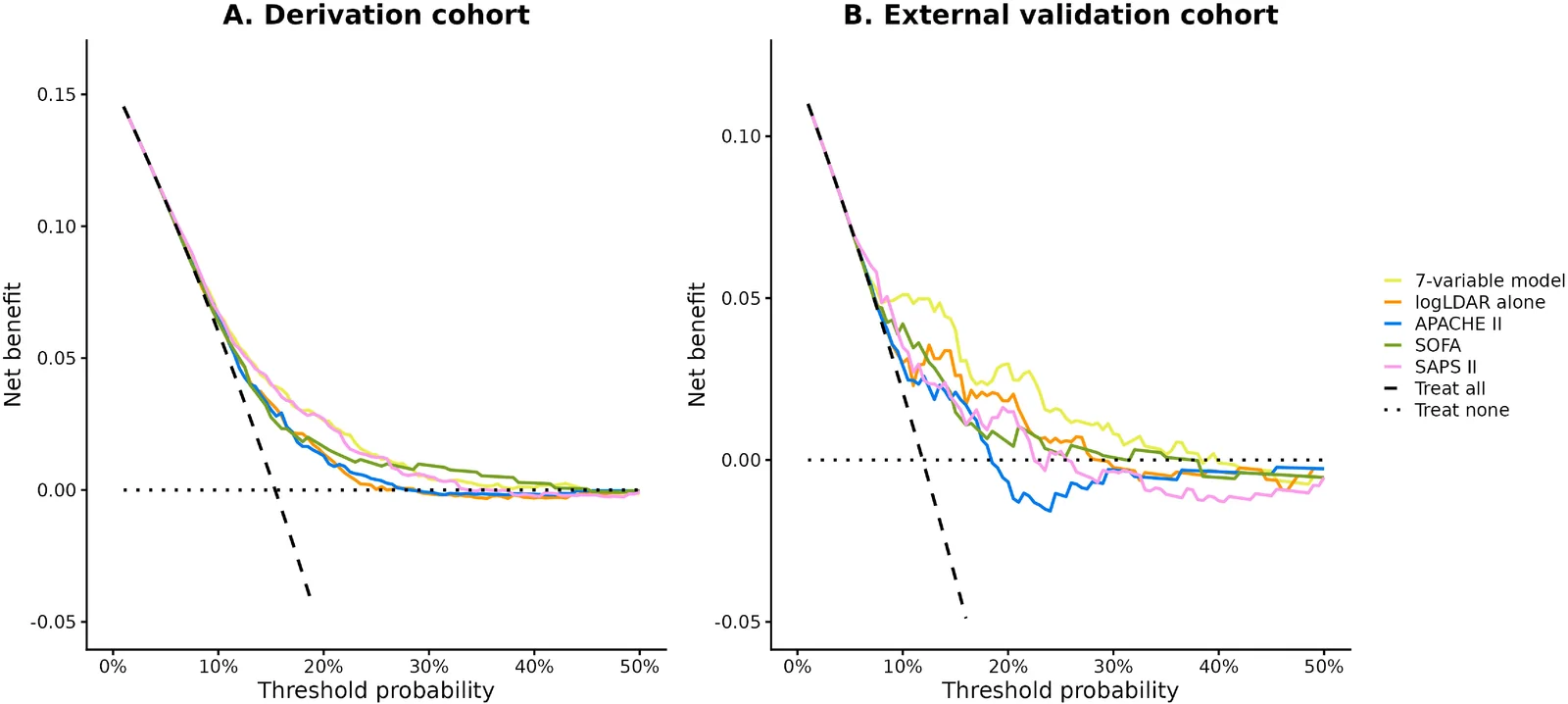
Decision curve analysis comparing net benefit of the 7-variable model, logLDAR alone, and conventional severity scores (APACHE II, SOFA, SAPS II) in **(A)** the derivation cohort and **(B)** the external validation cohort across a range of threshold probabilities.

## Discussion

Urosepsis represents a severe and increasingly prevalent complication of urinary tract infections, posing a substantial burden on patients and healthcare systems (Dreger et al., 2015; Ryan et al., 2020; Dickson et al., 2024; GBD 2021 Global Sepsis Collaborators, 2025; Morrissey et al., 2025). Although notable advances have been made in sepsis management, including the development of rapid diagnostic tools that shorten time to pathogen identification (Konjety and Chakole, 2024; Plata-Menchaca et al., 2024), most conventional severity scores remain complex, physiologically oriented, and challenging to apply rapidly in emergency settings. This complexity may lead to missed opportunities for early intervention in high-risk patients (Anagi et al., 2026; Lambden et al., 2019; Schoe et al., 2020). Consequently, there is an urgent clinical need for simpler, faster, and reproducible biomarkers to facilitate early risk stratification.

LDH and ALB are routine laboratory parameters that are readily available, cost-effective, and produce objective results. While each has been individually associated with poor outcomes in critical illness, a single marker cannot adequately capture the overall condition of the patient. LDAR integrates complementary pathophysiological information: LDH directly reflects tissue hypoxia, cellular necrosis, and metabolic derangement (Duman et al., 2016; Khan et al., 2020; Farhana and Lappin, 2023; Zhou et al., 2025; Ge et al., 2026), while ALB serves as a negative acute-phase reactant that reflects nutritional reserve, systemic inflammation, and hepatic synthetic function (Don and Kaysen, 2004; Jellinge et al., 2014; Morotti et al., 2017; Seidu et al., 2020; Linciano et al., 2022; Brahma et al., 2024). In the specific context of urosepsis, several unique mechanisms may enhance LDAR’s prognostic value. First, ascending infection causing acute pyelonephritis leads to direct renal tubular epithelial cell necrosis; LDH is abundantly present in renal tubular cells, and its elevation may directly reflect the extent of tubular damage specific to the urinary source. Second, urosepsis-associated AKI can impair the glomerular filtration barrier, leading to proteinuria and albumin loss—a mechanism distinct from the general inflammatory capillary leak seen in other sepsis sources. Third, post-renal obstruction (e.g., from stones, strictures, or prostatic enlargement) is a common precipitant of urosepsis; obstruction induces pressure nephropathy, affecting both LDH release (from pressure-induced cellular injury) and renal albumin handling. Finally, the progression from lower UTI to pyelonephritis to bacteremia involves escalating tissue damage, which may be reflected in a gradient of LDAR elevation. Thus, an elevated LDAR precisely identifies the high-risk clinical combination of “severe injury” and “poor reserve,” evaluating overall patient risk from a more comprehensive pathophysiological perspective.

In this dual-cohort study, we demonstrated for the first time, in a relatively large sample of critically ill patients with urosepsis, that elevated logLDAR is an independent risk factor for 28-day ICU and in-hospital mortality, exhibiting a linear dose-response relationship. The association was robust across multiple adjustment models, competing-risk analyses, and sensitivity analyses, and remained consistent across diverse patient subgroups. Moreover, adding logLDAR to six conventional severity scores significantly improved their predictive performance, providing direct evidence that this simple biomarker can effectively supplement existing scoring systems.

Clinically, the optimal logLDAR cut-off (7.010) showed moderate sensitivity (51.0%) but higher specificity (69.7%). This profile makes LDAR more suitable as an adjunctive rule-in tool rather than a standalone screening test. In critical care practice, where missing a high-risk patient carries significant consequences, LDAR should be used as an early-warning signal rather than a replacement for existing scores. It is particularly well-suited for a two-step strategy: first using LDAR for rapid risk stratification upon admission, followed by comprehensive assessment using conventional scores for precise prognostication. Moreover, LDAR requires only two routine biochemical tests, yields results within hours, needs no additional equipment or specialized training, and is broadly scalable—making it especially valuable in resource-limited primary hospitals or emergency departments as an auxiliary basis for ICU referral or early initiation of bundled treatment.

Compared with previous LDAR studies (Tu et al., 2026; Liang et al., 2023; Hu and Zhou, 2024), the present investigation offers several meaningful advancements that extend beyond mere statistical association toward clinical applicability. Most importantly, the dual-cohort design—internal MIMIC-IV for discovery and an independent external cohort for validation—substantially mitigates overfitting and single-center bias, enhancing both generalizability and the robustness of our evidence base. Our study is also the first to systematically quantify the incremental value of LDAR when added to six widely used severity scoring systems (APACHE II, APS III, Charlson, OASIS, SAPS II, and SOFA), directly comparing AUC improvements, NRI, and IDI across all scores. This provides concrete evidence that LDAR effectively supplements—rather than replaces—existing clinical tools. Furthermore, the integration of four machine learning algorithms for variable selection represents a more rigorous and transparent strategy than conventional stepwise regression. Equally important, we bridge the gap between statistical significance and clinical operability by identifying an optimal logLDAR cut-off (7.010) and proposing a pragmatic two-step strategy, adjunctive early-warning signal followed by precise assessment with conventional scores, offering clinicians a clear framework for bedside integration. Collectively, these advances position this study as a meaningful progression from prior work, moving LDAR evaluation from retrospective hypothesis generation toward a more clinically grounded evidence base.

## Limitations

Several limitations warrant consideration. First, the retrospective design, despite rigorous multiple imputation, cannot eliminate selection bias or unmeasured confounding. Key variables such as inflammatory cytokines (IL-6, PCT), detailed microbiological characteristics, and host genetic factors were unavailable, potentially limiting the predictive ceiling of our models. Second, LDAR’s sensitivity of only 51.0% precludes its use as a standalone confirmatory test; the 7-variable model, while outperforming most conventional scores, still yielded internal AUCs of only 0.674–0.677, indicating that important predictors remain unidentified. Third, the external validation cohort was relatively small (n=370) and single-center; although it reflects real-world practice, larger multi-center prospective studies are needed to confirm generalizability. Fourth, we used only baseline LDH and ALB values; dynamic changes at 48 or 72 hours might offer greater prognostic value and should be explored in future studies. Fifth, although multiple imputation assumed MAR, we cannot completely exclude bias from non-random missingness. Sixth, while we applied Fine-Gray models to address competing risks, standard Cox models may still overestimate absolute risk in the presence of competing events. Seventh, the differing case-finding strategies between cohorts (ICD-coded co-existing diagnoses in MIMIC-IV vs. culture-based clinical-microbiological criteria externally) may introduce non-differential misclassification, which should be considered when interpreting these findings. Eighth, although Model 3 included post-exposure interventions, the primary Model 2 estimates were consistent, mitigating concerns about mediator or collider bias, yet residual confounding from unmeasured treatment decisions persists. Finally, our logistic regression model, though interpretable, did not explore non-linear or deep-learning approaches. Future prospective interventional trials are needed to verify whether model-guided decisions truly improve patient outcomes.

## Conclusion

Using a dual retrospective design with an internal discovery cohort and an independent external validation cohort, this study systematically demonstrates that elevated logLDAR is an independent risk factor for 28-day ICU and in-hospital mortality in critically ill patients with urosepsis, with a linear dose-response relationship. Adding logLDAR to six conventional severity scores moderately improved their predictive performance. A machine learning-derived risk prediction model based on seven core variables, including logLDAR, showed comparable or moderately improved predictive ability relative to conventional scores, with acceptable cross-cohort stability. Although LDAR’s sensitivity as a standalone tool is limited, its simplicity, rapid availability, and reproducibility provide practical value as a useful supplement to existing scoring systems, particularly in resource-limited settings where rapid risk stratification is needed. Future prospective, multi-center studies with dynamic LDAR monitoring are warranted to validate its clinical utility and to evaluate whether LDAR-guided clinical decision pathways can truly improve outcomes in patients with urosepsis.

## Data Availability

The original contributions presented in the study are included in the article/**Supplementary Material**. Further inquiries can be directed to the corresponding author.

## Supplementary material

Supplementary Figure 1 Subgroup and interaction analyses (Model 2-adjusted) for the association between logLDAR (as a continuous variable) and 28-day ICU mortality. FDR = Benjamini-Hochberg false discovery rate for interaction P-values across 12 subgroups.

Supplementary Figure 2 Subgroup and interaction analyses (Model 2-adjusted) for the association between logLDAR (as a continuous variable) and 28-day hospital mortality. FDR = Benjamini-Hochberg false discovery rate for interaction P-values across 12 subgroups.
