# Supplementary material for: Association between lactate dehydrogenase-to-albumin ratio and short-term mortality in critically ill patients with urosepsis: evidence from dual retrospective cohorts
Source: Front Cell Infect Microbiol. 2026 Sep 16;16:1891664. doi: 10.3389/fcimb.2026.1891664 (PMC13623607; doi:10.3389/fcimb.2026.1891664)
Supplement: Supplementary file 1 [file Table1.docx]

**Table S1. List of abbreviations used in this study, organized by variable category.**

| **Abbreviation** | **Full term** |
| --- | --- |
| **Underlying diseases** | |
| AKI | acute kidney injury |
| CKD | chronic kidney disease |
| COPD | chronic obstructive pulmonary disease |
| DM | diabetes mellitus |
| HF | heart failure |
| HLD | hyperlipidemia |
| HPY | hypertension |
| IHD | ischemic heart disease |
| MI | myocardial infarction |
| **Vital signs** | |
| HR | heart rate |
| NBPD | non-invasive blood pressure diastolic |
| NBPS | non-invasive blood pressure systolic |
| RR | respiratory rate |
| SpO₂ | oxygen saturation |
| **Laboratory test results** | |
| ALB | albumin |
| ALT | alanine aminotransferase |
| AST | aspartate aminotransferase |
| Ca | calcium |
| Cl | chloride |
| CO₂CP | total carbon dioxide combining power |
| CREA | creatinine |
| GA | gap of anion |
| Glu | glucose |
| Hb | hemoglobin |
| HCT | hematocrit |
| INR | international normalized ratio |
| K | potassium |
| LAC | lactate |
| LDH | lactate dehydrogenase |
| Na | sodium |
| PCO₂ | partial pressure of carbon dioxide |
| PH | potential of hydrogen |
| PLT | platelet count |
| PO₂ | partial pressure of oxygen |
| PT | prothrombin time |
| PTT | partial thromboplastin time |
| RBC | red blood cell count |
| RDW | red cell distribution width |
| TB | total bilirubin |
| UREA | urea |
| WBC | white blood cell count |
| **Disease severity scores** | |
| APACHE II | Acute Physiology and Chronic Health Evaluation II |
| APS III | Acute Physiology Score III |
| OASIS | Oxford Acute Severity of Illness Score |
| SAPS II | Simplified Acute Physiology Score II |
| SOFA | Sequential Organ Failure Assessment |
| **Treatment measures** | |
| CRRT | continuous renal replacement therapy |
| GC | glucocorticoid |
| SA | sedation-analgesia |
| VP | vasopressor |
| **Exposure and outcomes** | |
| ICU | intensive care unit |
| LDAR | lactate dehydrogenase-to-albumin ratio |
| logLDAR | log₂-transformed LDAR |
| UTI | urinary tract infection |
| **Statistical and modeling terms** | |
| AUC | area under the curve |
| CI | confidence interval |
| CIF | cumulative incidence function |
| DCA | decision curve analysis |
| EPV | events-per-variable |
| FDR | false discovery rate |
| GBM | gradient boosting machine |
| HR | hazard ratio |
| IDI | integrated discrimination improvement |
| IQR | interquartile range |
| MAR | missing at random |
| NRI | net reclassification improvement |
| RCS | restricted cubic splines |
| ROC | receiver operating characteristic |
| sHR | subdistribution hazard ratio |
| SD | standard deviation |
| SQL | structured query language |
| SVM | support vector machine |
| VIF | variance inflation factor |
| **Other** | |
| IRB | institutional review board |
| MIMIC-IV | Medical Information Mart for Intensive Care IV |

**Table S2. Comparison of baseline characteristics between 28‑day ICU survivors and non‑survivors in the internal MIMIC‑IV cohort.**

|  | **ALL** | **survivor** | **Dead** | **P value** |
| --- | --- | --- | --- | --- |
|  | ***N=3060*** | ***N=2589*** | ***N=471*** |  |
| LDAR | 100 [72.7;153] | 96.7 [70.7;144] | 130 [87.4;220] | <0.001 |
| logLDAR | 6.84 (1.03) | 6.77 (0.98) | 7.25 (1.16) | <0.001 |
| Age | 70.5 (15.1) | 70.1 (15.2) | 73.0 (14.3) | <0.001 |
| Gender: | 1329 (43.4%) | 1129 (43.6%) | 200 (42.5%) | 0.681 |
| Race: | 1981 (64.7%) | 1698 (65.6%) | 283 (60.1%) | 0.025 |
| Weight | 82.0 (26.0) | 82.1 (25.9) | 81.4 (26.4) | 0.586 |
| HPY: | 1077 (35.2%) | 934 (36.1%) | 143 (30.4%) | 0.019 |
| AKI: | 1918 (62.7%) | 1554 (60.0%) | 364 (77.3%) | <0.001 |
| CKD: | 889 (29.1%) | 739 (28.5%) | 150 (31.8%) | 0.162 |
| DM: | 1155 (37.7%) | 995 (38.4%) | 160 (34.0%) | 0.074 |
| HLD: | 1074 (35.1%) | 944 (36.5%) | 130 (27.6%) | <0.001 |
| HF: | 1201 (39.2%) | 1001 (38.7%) | 200 (42.5%) | 0.133 |
| MI: | 316 (10.3%) | 249 (9.62%) | 67 (14.2%) | 0.003 |
| IHD: | 1126 (36.8%) | 948 (36.6%) | 178 (37.8%) | 0.664 |
| COPD: | 492 (16.1%) | 406 (15.7%) | 86 (18.3%) | 0.183 |
| SOFA | 6.90 (3.46) | 6.61 (3.27) | 8.48 (4.01) | <0.001 |
| APSIII | 58.9 (21.1) | 57.0 (20.1) | 69.6 (22.9) | <0.001 |
| SAPSII | 45.0 (13.4) | 43.8 (12.9) | 52.1 (13.9) | <0.001 |
| OASIS | 36.0 (8.33) | 35.5 (8.14) | 38.7 (8.85) | <0.001 |
| Charlson | 6.31 (2.91) | 6.16 (2.87) | 7.16 (2.99) | <0.001 |
| APACHEII | 21.8 (7.00) | 21.3 (6.88) | 24.5 (7.05) | <0.001 |
| HR | 91.5 (21.3) | 91.3 (21.3) | 92.4 (20.8) | 0.307 |
| NBPS | 120 (25.8) | 121 (25.8) | 116 (25.5) | <0.001 |
| NBPD | 67.6 (19.8) | 67.9 (19.6) | 65.9 (20.6) | 0.049 |
| RR | 20.2 (6.39) | 20.1 (6.40) | 20.4 (6.37) | 0.412 |
| Spo2 | 96.5 (4.68) | 96.6 (4.64) | 96.1 (4.89) | 0.064 |
| HCT | 31.3 (6.64) | 31.4 (6.62) | 30.6 (6.70) | 0.017 |
| Hb | 10.1 (2.20) | 10.2 (2.20) | 9.92 (2.19) | 0.023 |
| PLT | 200 (117) | 202 (116) | 190 (121) | 0.056 |
| RDW | 16.1 (2.61) | 15.9 (2.51) | 16.9 (3.03) | <0.001 |
| RBC | 3.40 (0.78) | 3.42 (0.77) | 3.30 (0.80) | 0.004 |
| WBC | 13.9 (14.8) | 13.5 (13.9) | 15.9 (18.6) | 0.009 |
| ALB | 2.90 (0.62) | 2.93 (0.60) | 2.75 (0.65) | <0.001 |
| GA | 15.7 (4.76) | 15.6 (4.71) | 16.3 (4.99) | 0.003 |
| Ca | 8.26 (0.98) | 8.27 (0.98) | 8.22 (0.99) | 0.291 |
| Cl | 104 (8.02) | 104 (7.86) | 104 (8.86) | 0.203 |
| Glu | 133 [105;175] | 133 [106;175] | 134 [100;176] | 0.180 |
| K | 4.23 (0.81) | 4.21 (0.80) | 4.31 (0.84) | 0.016 |
| Na | 139 (6.86) | 139 (6.74) | 138 (7.48) | 0.483 |
| CO2CP | 23.9 (6.55) | 24.1 (6.56) | 22.9 (6.38) | <0.001 |
| LAC | 2.40 (1.99) | 2.33 (1.96) | 2.77 (2.08) | <0.001 |
| PCO2 | 42.2 (12.7) | 42.3 (12.7) | 41.7 (12.6) | 0.379 |
| PH | 7.35 (0.11) | 7.35 (0.10) | 7.33 (0.11) | 0.002 |
| PO2 | 116 (95.3) | 118 (96.7) | 106 (87.2) | 0.008 |
| INR | 1.40 [1.20;1.70] | 1.30 [1.20;1.70] | 1.50 [1.20;2.00] | <0.001 |
| PT | 15.0 [13.0;18.9] | 14.8 [12.9;18.5] | 16.5 [13.8;22.0] | <0.001 |
| PTT | 32.4 [27.8;41.2] | 32.1 [27.6;40.3] | 35.0 [28.9;47.0] | <0.001 |
| ALT | 25.0 [15.0;53.0] | 25.0 [15.0;52.0] | 28.0 [17.0;62.5] | 0.002 |
| AST | 39.0 [23.0;86.0] | 38.0 [22.0;81.0] | 51.0 [27.0;114] | <0.001 |
| TB | 0.70 [0.40;1.40] | 0.60 [0.40;1.30] | 0.90 [0.40;2.70] | <0.001 |
| CREA | 1.30 [0.80;2.20] | 1.30 [0.80;2.20] | 1.50 [0.90;2.40] | 0.006 |
| UREA | 29.0 [18.0;49.0] | 28.0 [17.0;48.0] | 36.0 [22.0;57.0] | <0.001 |
| LDH | 284 [214;409] | 278 [210;396] | 348 [247;546] | <0.001 |
| SA: | 2052 (67.1%) | 1685 (65.1%) | 367 (77.9%) | <0.001 |
| VP: | 1908 (62.4%) | 1537 (59.4%) | 371 (78.8%) | <0.001 |
| GC: | 995 (32.5%) | 809 (31.2%) | 186 (39.5%) | 0.001 |
| Ventilation: | 2637 (86.2%) | 2228 (86.1%) | 409 (86.8%) | 0.705 |
| CRRT: | 321 (10.5%) | 235 (9.08%) | 86 (18.3%) | <0.001 |

**Table S3. Comparison of baseline characteristics between 28-day ICU survivors and non-survivors in the external validation cohort (Xingyi People's Hospital Affiliated to Guizhou Medical University).**

|  | **ALL** | **Survivor** | **Dead** | **P value** |
| --- | --- | --- | --- | --- |
|  | ***N=370*** | ***N=326*** | ***N=44*** |  |
| LDAR | 96.1 [68.6;140] | 91.5 [66.5;132] | 137 [113;270] | <0.001 |
| logLDAR | 6.78 (1.07) | 6.68 (1.02) | 7.50 (1.16) | <0.001 |
| Age | 70.4 (15.2) | 70.0 (15.4) | 73.1 (13.6) | 0.174 |
| Gender: | 162 (43.8%) | 138 (42.3%) | 24 (54.5%) | 0.170 |
| Weight | 66.4 (11.2) | 66.4 (11.4) | 66.3 (9.53) | 0.964 |
| HPY: | 128 (34.6%) | 116 (35.6%) | 12 (27.3%) | 0.358 |
| AKI: | 221 (59.7%) | 183 (56.1%) | 38 (86.4%) | <0.001 |
| CKD: | 107 (28.9%) | 90 (27.6%) | 17 (38.6%) | 0.181 |
| DM: | 145 (39.2%) | 126 (38.7%) | 19 (43.2%) | 0.679 |
| HLD: | 129 (34.9%) | 115 (35.3%) | 14 (31.8%) | 0.777 |
| HF: | 137 (37.0%) | 120 (36.8%) | 17 (38.6%) | 0.945 |
| MI: | 28 (7.57%) | 22 (6.75%) | 6 (13.6%) | 0.124 |
| IHD: | 133 (35.9%) | 116 (35.6%) | 17 (38.6%) | 0.819 |
| COPD: | 58 (15.7%) | 55 (16.9%) | 3 (6.82%) | 0.133 |
| SOFA | 6.68 (3.51) | 6.40 (3.41) | 8.70 (3.66) | <0.001 |
| APSIII | 59.0 (22.1) | 57.5 (21.7) | 70.3 (21.7) | 0.001 |
| SAPSII | 45.0 (13.9) | 44.0 (13.8) | 52.4 (12.7) | <0.001 |
| OASIS | 36.8 (8.77) | 35.9 (8.45) | 44.2 (7.69) | <0.001 |
| Charlson | 6.13 (3.15) | 5.95 (3.05) | 7.45 (3.55) | 0.010 |
| APACHEII | 21.6 (7.20) | 21.2 (7.26) | 24.3 (6.13) | 0.003 |
| HR | 92.2 (20.9) | 91.8 (20.9) | 95.6 (21.0) | 0.256 |
| NBPS | 119 (25.2) | 120 (25.2) | 114 (25.0) | 0.111 |
| NBPD | 66.2 (19.0) | 66.3 (18.9) | 65.2 (19.7) | 0.731 |
| RR | 19.8 (6.08) | 20.0 (6.18) | 18.5 (5.16) | 0.076 |
| Spo2 | 96.6 (4.03) | 96.6 (4.02) | 96.6 (4.13) | 0.962 |
| HCT | 31.1 (6.26) | 31.1 (6.20) | 31.7 (6.77) | 0.548 |
| Hb | 10.1 (2.08) | 10.1 (2.06) | 10.3 (2.29) | 0.538 |
| PLT | 182 [121;265] | 184 [125;268] | 153 [87.5;226] | 0.061 |
| RDW | 16.0 (2.39) | 15.8 (2.21) | 16.9 (3.34) | 0.054 |
| RBC | 3.40 (0.74) | 3.40 (0.72) | 3.42 (0.88) | 0.846 |
| WBC | 11.2 [7.73;17.0] | 10.8 [7.23;16.3] | 13.4 [11.2;19.4] | 0.002 |
| ALB | 2.93 (0.63) | 2.94 (0.62) | 2.89 (0.71) | 0.658 |
| GA | 16.0 (4.81) | 15.6 (4.66) | 18.8 (5.00) | <0.001 |
| Ca | 8.23 (0.99) | 8.23 (1.02) | 8.21 (0.79) | 0.895 |
| Cl | 104 (8.24) | 104 (7.99) | 103 (9.98) | 0.810 |
| Glu | 135 [104;170] | 134 [103;174] | 144 [106;169] | 0.697 |
| K | 4.26 (0.83) | 4.25 (0.84) | 4.40 (0.79) | 0.229 |
| Na | 138 (7.55) | 138 (7.17) | 138 (10.0) | 0.942 |
| CO2CP | 23.5 (6.27) | 24.0 (6.26) | 20.1 (5.35) | <0.001 |
| LAC | 2.26 (1.88) | 2.15 (1.78) | 3.12 (2.34) | 0.011 |
| PCO2 | 41.4 (11.6) | 41.8 (11.8) | 38.5 (9.46) | 0.039 |
| PH | 7.35 (0.10) | 7.35 (0.10) | 7.31 (0.11) | 0.020 |
| PO2 | 120 (104) | 121 (106) | 111 (88.7) | 0.505 |
| INR | 1.40 [1.20;1.70] | 1.30 [1.20;1.70] | 1.50 [1.20;2.08] | 0.053 |
| PT | 14.9 [12.9;18.6] | 14.8 [12.8;18.2] | 16.4 [13.3;22.6] | 0.076 |
| PTT | 32.4 [27.8;39.4] | 32.0 [27.7;39.2] | 35.6 [29.2;46.6] | 0.086 |
| ALT | 26.0 [13.0;56.0] | 25.0 [13.0;53.5] | 32.0 [19.8;60.2] | 0.063 |
| AST | 37.0 [21.0;85.0] | 35.0 [20.0;81.0] | 62.5 [30.8;145] | 0.001 |
| TB | 0.70 [0.40;1.37] | 0.65 [0.40;1.30] | 0.85 [0.40;3.03] | 0.055 |
| CREA | 1.30 [0.80;2.20] | 1.30 [0.80;2.10] | 1.85 [1.20;3.70] | <0.001 |
| UREA | 28.5 [17.0;51.0] | 27.0 [16.0;47.8] | 45.0 [32.8;78.2] | <0.001 |
| LDH | 274 [212;375] | 254 [205;358] | 370 [306;629] | <0.001 |
| SA: | 228 (61.6%) | 197 (60.4%) | 31 (70.5%) | 0.263 |
| VP: | 204 (55.1%) | 168 (51.5%) | 36 (81.8%) | <0.001 |
| GC: | 111 (30.0%) | 94 (28.8%) | 17 (38.6%) | 0.247 |
| Ventilation: | 317 (85.7%) | 277 (85.0%) | 40 (90.9%) | 0.409 |
| CRRT: | 34 (9.19%) | 27 (8.28%) | 7 (15.9%) | 0.158 |

**Table S4: Variance inflation factors (VIF) for all variables in Cox Model 3.**

| **Variable** | **VIF** | **Variable** | **VIF** |
| --- | --- | --- | --- |
| logLDAR | 1.56 | Age | 1.52 |
| Gender | 1.16 | Race | 1.08 |
| Weight | 1.27 | AKI | 1.19 |
| CKD | 1.30 | HLD | 1.06 |
| HF | 1.29 | MI | 1.11 |
| COPD | 1.08 | NBPS | 1.85 |
| NBPD | 1.74 | Spo2 | 1.09 |
| RBC | 1.18 | WBC | 1.06 |
| GAP | 2.04 | K | 1.20 |
| TCO2 | 1.70 | LAC | 1.89 |
| PH | 1.47 | PO2 | 1.13 |
| PTT | 1.09 | ALT | 1.38 |
| TB | 1.45 | CREA | 2.08 |
| SA | 1.24 | VP | 1.33 |
| GC | 1.11 | CRRT | 1.30 |

Note: VIF = variance inflation factor. All VIF values are below 5, confirming no significant multicollinearity. Maximum VIF = 2.08 (creatinine). SA = sedation-analgesia therapy.
